# On the Use of Carbonate of Potash in Diseases of the Urinary Organs. In a Letter from M. Guyton Morveau to the Editors of the Annales de Chimie

**Published:** 1810-01

**Authors:** 


### *On the Use of Carbonate of Potash in Diseases of the Urinary Organs. In a Letter from* M. GUYTON MORVEAU *to the Editors of the Annales de Chimie.*

I Have recently met with a Memoir, published in the eleventh volume of the Transactions of the Italian Society of Sciences, wherein M. Mascagni gives an account of an experiment he made on himself with a salt, which I never heard of having been administered internally, at least in such large doses as he mentions. I shall therefore present you with a translation of his paper, accompanied by such observations as have occurred to me.

"Some years previous to 1799, (as Mascagni informs us) I was subject to pains in the lumbar region, and voided from time to time small calculi of an ochry yellow or brick colour. Knowing that gaseous alkaline water had been used with some success, I took it several times, and found myself much better.

"It afterwards occurred to me, that I might obtain even better effects from carbonate of potash. In October 1798, I exposed a concentrated solution of potash to the action of carbonic acid, procured from grapes in a state of fermentation; and in this way I obtained a stock of carbonate of potash, well saturated and crystalized.

"In the months of August and September, 1799, having been compelled to lead a sedentary life, I was seized with violent pains in the kidnies, and I voided a considerable quantity of gravelly concretions, some of which, on account of their weight, might be considered as true calculi. They were reddish and crystalized, and were deposited sited at the bottom of the vessel every time that I made water. The shining faces of these calculi could be distinguished through the liquid, which was a little turbid, but nevertheless transparent. I was also subject to a superabundance of acid in the stomach, which was perceived in the mouth.

"I examined my urine, and found a free acid, which declared itself by reddening turnsole paper. This induced me to suppose, that my gravel stones might have been formed by the lithic acid, which was in excess in my urine. These concretions, when well washed and dried on bibulous paper, then soaked in water and placed on turnsole paper, also reddened it. When subjected to the action of distilled water, there was a solution in the proportions known with respect to the lithic acid. When afterwards treated in any other manner, they gave the most unequivocal proofs of the presence of this acid."

Here I must observe, that what M. Mascagni calls lithic acid is the same with the uric acid of Fourcroy and Pearson. There is reason to regret, that M. Mascagni has not stated at greater length, the processes by which he ascertained the nature of this acid. It results, indeed, from the great work of Messrs. Fourcroy and Vauquelin, on human urine, that it contains uric acid, and that the red crystals deposited by it are thus formed. But they ascertained at the same time, that its peculiar acidity is not great, and must contribute but in a very small degree to give the urine its acid character. They have besides, confirmed the observations of Scheele, as to the presence of free phosphoric acid in the same acid. It would have been easy for M. Mascagni to put his conclusion beyond the reach of objection, were it only for the property which the last of these acids has of being fixed in a strong heat, while the former is speedily resolved into its elements.

"Having been thus convinced (continues M. Mascagni) of the nature of this acid, I resolved to make use of carbonate of potash, and to watch the results. The first day I took a whole drachm, one half before breakfast and the other half at bed-time; I had dined at one o'clock. This salt, dissolved in ten ounces of water, had very little taste; it did not cause the least alteration in the stomach nor intestines: but the instant the solution was introduced into the stomach, it occasioned an extrication of carbonic acid, which was felt in the mouth as coming from the œsophagus; a part afterwards passed off by the anus: a proof of the combination of potash with another acid, which must have driven off the carbonic acid.

"On the second day I raised the dose to two drachms; on the third to three; and I continued this last dose for ten days, making the solution in 20 ounces of water."

We find from the above, that in the space of ten days M. Mascagni must have taken internally 29 grammes, or nearly three ounces of carbonate of potash.

"I had remarked, as I have before mentioned, that my urine reddened turnsole paper; I subjected to the same experiment what I now voided, and I perceived from the time I began to use the saline solution, a diminution in the intensity of the colour. On the second day the turnsole paper exhibited almost no change; on the third day there was none at all; a proof that the acid had been saturated. My pains also nearly went off on the third day, and I voided no more calculi with my urine. Afterwards the pains ceased entirely, my urine was less turbid, and I ascertained the presence of potash in excess from the red garnet colour assumed by paper died yellow by turmeric, as well as by other substances, which, when saturated by potash, formed neutral salts.

"I then gave up the use of carbonate of potash, and I was several months without voiding any calculi. Having been since attacked with this disease, I had recourse to the same remedy, and obtained the same effects. I repeated this medico-chemical experiment on every occasion that I felt the same inconvenience, and always with the like success. It is now two years since I voided any calculi, although I no longer make use of carbonate of potash.

"These experiments evidently prove, that potash is introduced into the urinary passages; that it saturates the lithic acid; and that on forming with it a more soluble neutral salt, it opposes the production of concretions, of which calculi like those I have described are formed. There may, without doubt, be concretions of another nature; and it remains to be proved if the same treatment will answer every description of calculi."

The above Memoir of M. Mascagni is terminated by observations on the action which the alkalies exercise in general on all animal concretions, and on the advantages which might result from their use in diseases of the lungs, and other analogous affections. He gives examples of the happy effects of this treatment, particularly in an epidemic disease which had made great ravages in the province of Vienna, and where he succeeded beyond all expectation.

*February* 27, 1809.

